# Management of Rhabdomyolysis Complicated by Acute Kidney Injury Using Fluid Therapy and Mannitol: A Case Report

**DOI:** 10.7759/cureus.114180

**Published:** 2026-08-08

**Authors:** Italo Gibran Reyes Rosas, Maite Bravo Vidal

**Affiliations:** 1 Intensive Care Unit, Hospital Regional de Poza Rica, Poza Rica, MEX; 2 Emergency Department, Hospital General De Zona No. 24, Instituto Mexicano del Seguro Social (IMSS), Poza Rica, MEX

**Keywords:** acute kidney injury, creatine kinase, crystalloid therapy, mannitol, myoglobin, rhabdomyolysis, skeletal muscle injury

## Abstract

Rhabdomyolysis is a potentially life-threatening condition that may be complicated by acute kidney injury (AKI), requiring prompt recognition and early treatment to prevent adverse outcomes. We report the case of a young female patient who developed rhabdomyolysis complicated by AKI. Initial laboratory evaluation demonstrated a peak creatine kinase (CK) level of 3,976 U/L, together with biochemical evidence of AKI. The patient was managed with aggressive intravenous crystalloid therapy, close monitoring of urine output and renal function, and adjunctive low-dose mannitol after individualized clinical assessment. During hospitalization, she showed progressive clinical improvement, recovery of urine output, normalization of renal function, and a marked decline in CK levels without requiring renal replacement therapy. She was discharged in stable condition with complete clinical recovery. This case highlights the importance of early recognition, prompt fluid resuscitation, and individualized management of rhabdomyolysis complicated by AKI while emphasizing that the favorable outcome observed in this patient does not establish a causal benefit of adjunctive mannitol.

## Introduction

Rhabdomyolysis is characterized by skeletal muscle injury and necrosis with release of intracellular constituents, including creatine kinase (CK), electrolytes, and myoglobin, into the circulation [1,2].

Acute kidney injury (AKI) is among its most serious systemic complications and results from a combination of intrarenal vasoconstriction, direct tubular toxicity, tubular obstruction by pigment casts, and volume depletion [1,3].

The optimal strategy for preventing or treating rhabdomyolysis-associated AKI remains controversial. Current evidence supports early intravenous crystalloid resuscitation, whereas routine urine alkalinization with sodium bicarbonate and routine administration of mannitol are not supported by sufficiently robust clinical evidence [2,4-6].

We report the case of a young female patient with rhabdomyolysis complicated by AKI who was managed with intravenous crystalloid therapy and mannitol and subsequently demonstrated favorable clinical and biochemical outcomes.

## Case presentation

A 22-year-old previously healthy woman presented to the emergency department with severe bilateral lower-extremity pain, progressive muscle weakness, and inability to ambulate normally. She denied any significant past medical history, chronic medication use, or previous episodes of muscle disease. One week before symptom onset, she had performed more than two hours of high-intensity functional exercise after approximately three years of physical inactivity.

Initially, she developed severe pain in the left lower extremity associated with difficulty standing and walking. She self-medicated with diclofenac and a vitamin B complex preparation without symptom relief. The following day, she sought medical attention at a primary care facility, where intramuscular ketorolac (30 mg) provided only partial improvement, prompting referral to the emergency department.

On admission, she reported severe bilateral lower-extremity pain (9/10 on the Numeric Rating Scale) associated with generalized muscle weakness. Initial laboratory investigations demonstrated evidence of rhabdomyolysis with elevated muscle enzymes, including CK of 2,217 U/L, aspartate aminotransferase (AST) of 901 U/L, alanine aminotransferase (ALT) of 203 U/L, lactate dehydrogenase (LDH) of 1,505 U/L, and CK-MB of 426 U/L. The elevated CK-MB level was interpreted in the context of extensive skeletal muscle injury. Cardiac-specific biomarkers, including troponin, were not obtained; therefore, concomitant myocardial injury could not be definitively excluded. This represents a limitation of the cardiac evaluation in this case. Intravenous 0.9% normal saline was initiated together with analgesic therapy.

Despite aggressive intravenous hydration, urine output progressively declined to approximately 0.5 mL/kg/hour for 18 hours, fulfilling Kidney Disease: Improving Global Outcomes (KDIGO) stage 1 criteria for AKI based on oliguria. In this case, AKI was identified based on the urine-output criterion. Urinalysis demonstrated acidic urine with mild proteinuria, abundant leukocytes, bacteria, and mucus filaments. No urine culture was obtained, and no antibiotic therapy was administered. Therefore, a concomitant urinary tract infection could not be definitively confirmed or excluded, and sample contamination also remained a possibility. Because the target urine output of at least 1.5 mL/kg/hour was not achieved, intravenous fluid therapy was changed to lactated Ringer’s solution, and intravenous mannitol (37 g every 24 hours) was initiated.

Following the initial treatment period and transient biochemical improvement, mannitol was discontinued after reassessment of the therapeutic strategy, while intravenous hydration was continued. Thereafter, the patient developed worsening laboratory parameters and persistently inadequate urine output. However, the patient subsequently demonstrated worsening laboratory parameters, including an increase in CK to 3,976 U/L, persistent elevation of AST, serum creatinine of 1.7 mg/dL providing subsequent biochemical evidence of AKI, and blood urea nitrogen of 50 mg/dL, accompanied by persistently inadequate urine output. Intravenous fluids were intensified, and furosemide (2 mg/kg/day) was administered as a rescue attempt to improve persistent oliguria rather than as routine prophylaxis for pigment nephropathy. No significant improvement in urine output or biochemical markers was observed following its administration.

Given the inadequate response, treatment was modified by reintroducing intravenous lactated Ringer’s solution (50 mL/kg/day) combined with intravenous mannitol (37 g every 24 hours). Within 24 hours, urine output increased markedly to 5.5 mL/kg/hour, serum creatinine normalized to 0.7 mg/dL, blood urea nitrogen decreased to 40 mg/dL, and urinalysis no longer demonstrated proteinuria or myoglobinuria. Progressive reduction in muscle enzyme levels was subsequently observed.

The patient continued to improve clinically, with complete resolution of lower-extremity pain and recovery of muscle strength. By the end of hospitalization, urine output remained adequate, CK had decreased to 907 U/L, and renal function had completely recovered. She was discharged in stable condition with recommendations for adequate oral hydration, avoidance of strenuous physical activity until outpatient follow-up, and gradual return to normal daily activities.

Serial laboratory values obtained throughout hospitalization are summarized in Table 1.

**Table 1:** Serial laboratory values during hospitalization. Reference ranges are provided for comparison. Exact numerical laboratory values were not available for all time points; therefore, qualitative trends are reported when objective numerical data were unavailable. No values were estimated or inferred.  Laboratory data are presented according to the clinically documented therapeutic phases. Exact hospital-day laboratory values were not available for all treatment phases; therefore, additional time points could not be reliably reconstructed. Serum potassium, phosphate, and calcium values were not available in the clinical records reviewed; therefore, these parameters could not be reported. This represents a limitation of the present case report.

Laboratory Parameter	Reference Range	Admission	Clinical Deterioration	After Reintroduction of Mannitol	Discharge
Creatine kinase (CK), U/L	26–192	2,217	3,976	Decreasing	907
Aspartate aminotransferase (AST), U/L	10–40	901	Persistently elevated	Decreasing	Improved
Alanine aminotransferase (ALT), U/L	7–56	203	—	Decreasing	Improved
Lactate dehydrogenase (LDH), U/L	140–280	1,505	—	Decreasing	Improved
CK-MB, U/L	<25	426	—	—	—
Serum creatinine, mg/dL	0.5–1.1	Normal	1.7	0.7	0.7
Blood urea nitrogen (BUN), mg/dL	7–20	—	50	40	Improved
Urine output, mL/kg/h	Target ≥1.5	Declining	~0.5 for 18 h	5.5	Adequate

## Discussion

In the present case, initial conventional therapy did not result in a significant reduction in CK levels or improvement in the AKI that developed early during the patient's clinical course. The diagnosis was based primarily on reduced urine output; AKI may be diagnosed by urine-output criteria even in the absence of a marked increase in serum creatinine [7].

Although the peak CK level in this patient was relatively modest (3,976 U/L), AKI can occur at lower CK concentrations, as the risk of kidney injury is influenced not only by the magnitude of CK elevation but also by factors such as volume status, timing of presentation, concomitant nephrotoxic exposures, and individual susceptibility [2].

Treatment was therefore modified to include low-dose mannitol in combination with a lactated crystalloid solution. This statement describes the therapeutic course of the present patient and should not be interpreted as evidence of superiority over crystalloid therapy alone.

Lactated Ringer's solution was selected to reduce chloride exposure. Administration of large volumes of 0.9% saline can produce hyperchloremic metabolic acidosis, whereas balanced crystalloids contain lower chloride concentrations and have been associated with fewer adverse kidney outcomes in broad hospitalized populations [8,9]. Evidence specific to rhabdomyolysis, however, is insufficient to establish one crystalloid as universally superior [2,6].

The patient had also been exposed to nonsteroidal anti-inflammatory drugs (NSAIDs), including self-administered diclofenac and intramuscular ketorolac before admission. NSAIDs may impair renal perfusion through inhibition of prostaglandin-mediated renal vasodilation, particularly in the setting of volume depletion. Therefore, NSAID exposure may have contributed to the development of AKI in this patient, although its specific contribution cannot be determined from this single case [10].

Mannitol produces osmotic diuresis and has theoretical benefits that include increased urinary flow, dilution of tubular myoglobin, and reduction of tissue edema. Nevertheless, clinical evidence demonstrating prevention of AKI, dialysis, or mortality with routine mannitol use is limited and inconsistent [1,4-6].

Loop diuretics may increase urine output but do not correct intravascular hypovolemia. Their routine use to prevent rhabdomyolysis-associated AKI is not recommended. Acidic urine may favor myoglobin precipitation in the renal tubules, whereas urinary alkalinization with bicarbonate has historically been proposed as a preventive strategy; however, evidence supporting its routine use remains insufficient [1,2,5].

Following the introduction of lactated Ringer's solution and low-dose mannitol, the patient demonstrated progressive clinical improvement, recovery of urine output, normalization of renal function, and a marked decline in muscle enzyme levels. Because this is a single case and the interventions were administered together, a causal effect attributable specifically to mannitol cannot be established. An alternative explanation is that the observed improvement was primarily related to optimization of intravenous crystalloid therapy rather than to mannitol itself. Because lactated Ringer’s solution and mannitol were administered concurrently, their individual contributions to the subsequent clinical and biochemical improvement cannot be distinguished.

Current guidelines and systematic reviews conditionally recommend early intravenous fluid resuscitation but recommend against routine bicarbonate or mannitol therapy because the certainty of evidence is low and available studies are predominantly retrospective or observational [4-6]. The selective use of mannitol may therefore be considered only after individualized assessment and confirmation of adequate intravascular volume, with close monitoring of urine output and kidney function [1,5]. In the present case, serum osmolality was not available in the clinical record, which represents a limitation of the reported monitoring [1,5].

## Conclusions

This case highlights the successful management of severe rhabdomyolysis complicated by AKI through early recognition, aggressive intravenous fluid resuscitation, and carefully selected adjunctive therapy with mannitol after adequate volume replacement. Prompt diagnosis, close monitoring of renal function, electrolytes, urine output, and CK levels, together with timely supportive care, were essential for achieving complete renal recovery without the need for renal replacement therapy.

Although the role of mannitol remains controversial and should be individualized, this case suggests that it may be considered in carefully selected patients after adequate fluid resuscitation. Early multidisciplinary management and individualized treatment strategies remain fundamental to improving outcomes in patients with severe rhabdomyolysis and AKI.
